# Supplementary material for: Decoding the 5′ nucleotide bias of PIWI-interacting RNAs
Source: Nat Commun. 2019 Feb 19;10:828. doi: 10.1038/s41467-019-08803-z (PMC6381166; doi:10.1038/s41467-019-08803-z)
Supplement: Supplementary file 1 — Supplementary Information [file 41467_2019_8803_MOESM1_ESM.pdf]

Supplementary Information

Decoding the 5' nucleotide bias of PIWI-interacting RNAs (piRNAs)

Stein, Genzor, Mitra, Elchert et al.

Supplementary Figures (1-5) and Data (1-6).

Supplementary Figures

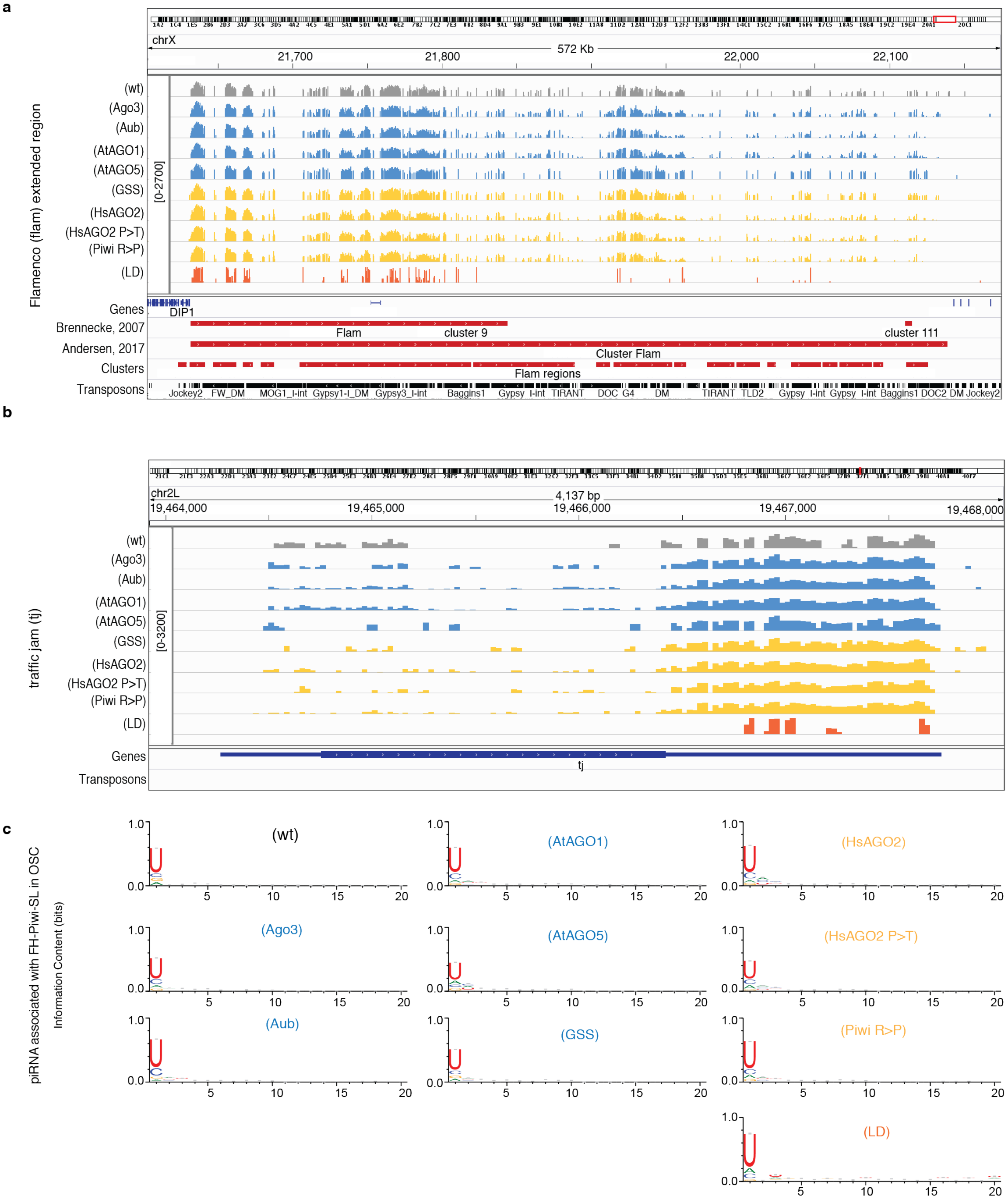

Supplementary Fig. 1. Characterization of piRNAs associated with FH-Piwi-SL(wt) and SL mutants in OSC. Genome tracks (IGV) of unique mapping reads associated with the indicated Piwi protein showing the extended flamenco (flam) cluster region **a**, and traffic jam (tj) **b**. **c,** Sequence Logo of piRNAs associated with the indicated Piwi-SL protein. [M=1: Unique mapping reads are considered]

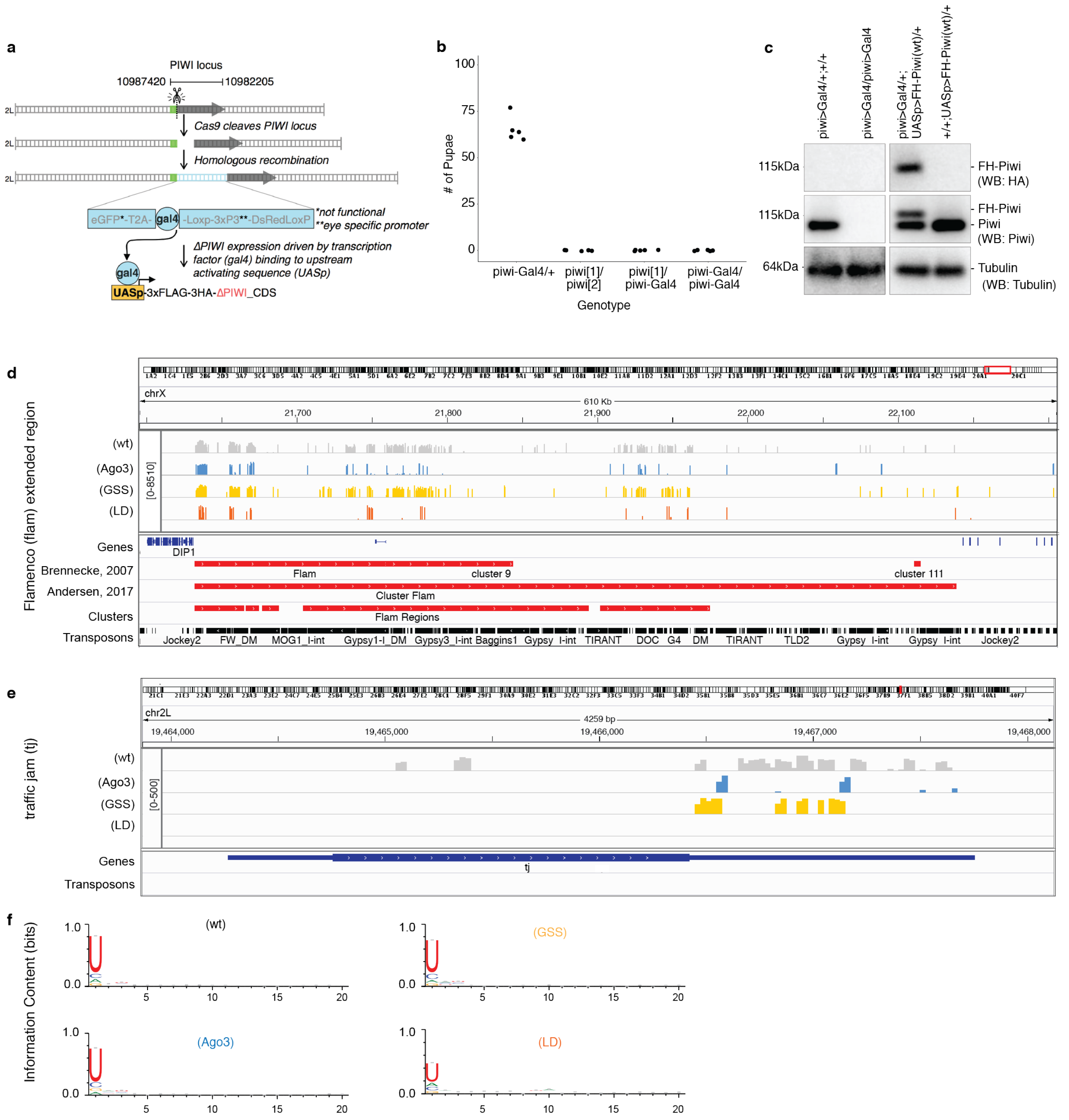

Supplementary Fig. 2. A genetic model to assess the function of mutations in *piwi*, and characterization of Piwi-piRNAs in *Dm* ovaries. a The design of a genetic rescue system for *piwi* function: We used CRISPR/Cas to insert a Gal4 open reading frame (ORF) and a selection cassette between the *piwi* promoter and *piwi*'s coding sequence (cds). The mutant allele expresses Gal4 driven by the *piwi* promoter instead of *piwi*. Rescue constructs expressing either wt Flag-HA(FH)-tagged Piwi or SL mutants are introduced as transgenes under the control of Upstream Activating Sequence (UAS). **b** Fertility of *Piwi*>Gal4 homozygous flies is compared to the classical *piwi1* and *piwi2* alleles in individual crosses. **c** *Piwi*>Gal4, UAS_Piwi heterozygous flies expresses FH-Piwi to similar levels as the endogenous Piwi. Genome tracks (IGV) of unique mapping reads associated with the indicated Piwi protein showing the

extended flamenco (flam) cluster region **d**, and traffic jam (tj) **e**. **f** Sequence Logo of piRNA associated with the indicated Piwi-SL protein. [M=1: Unique mapping reads are considered]

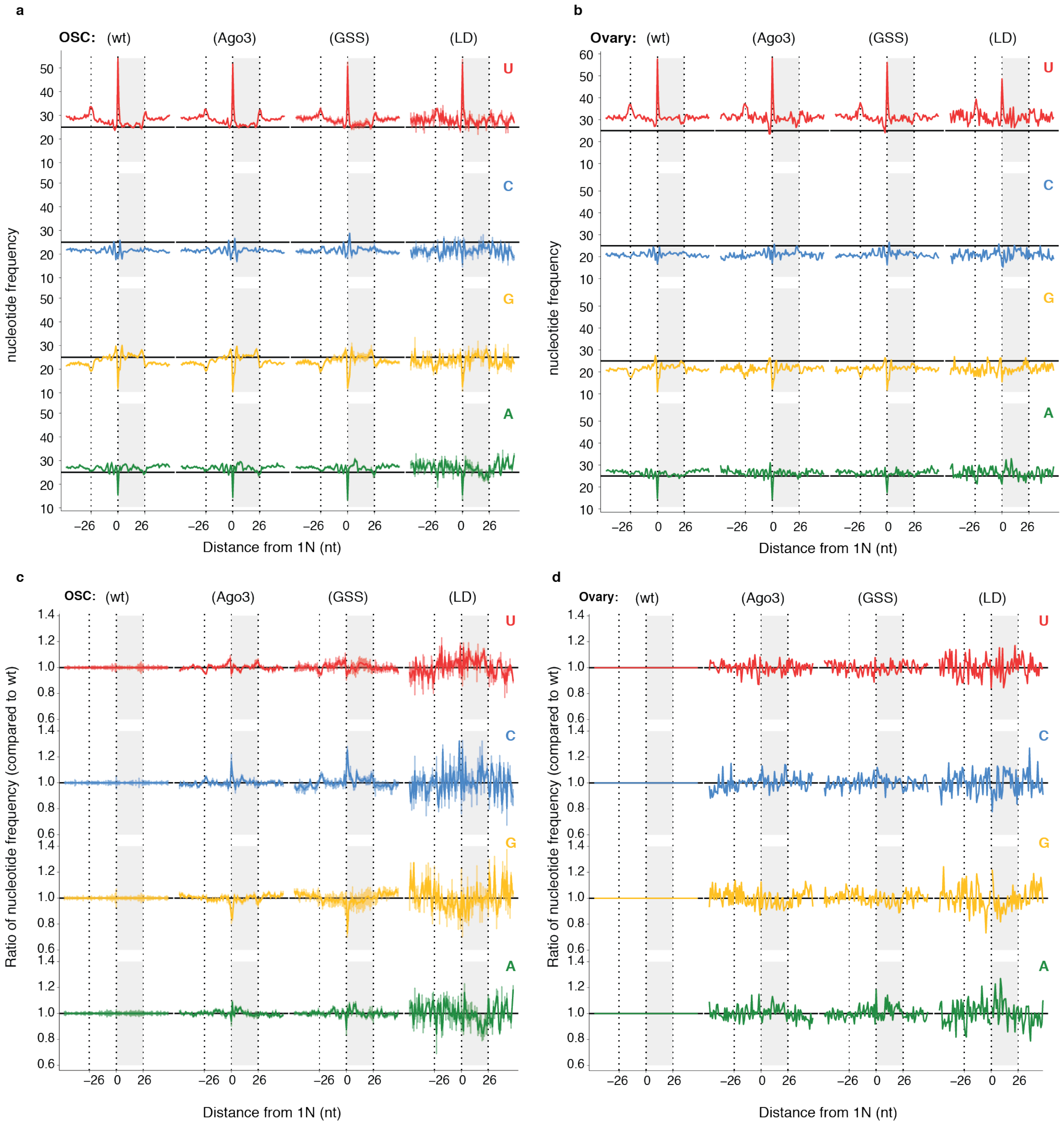

Supplementary Fig. 3. Sequence preferences at the piRNA precursor level. Individual nucleotide frequencies acquired from metagene analysis for a 101 nt window surrounding the 5' end of Piwi-piRNAs (position 0) in OSC [n = 3] **a** and fly Ovary [n = 1] **b.** The horizontal line represents the genomic nucleotide average of 25%. piRNA-generating regions have above the genomic average U and A content, and are below the genomic average in the G and C content in both OSC and fly Ovaries, which remains largely unchanged in SL mutants. Ratio of metagene-based nucleotide frequencies in SL-mutants compared to (wt) in OSC [n = 3] **c** and fly Ovaries [n = 1] **d.**

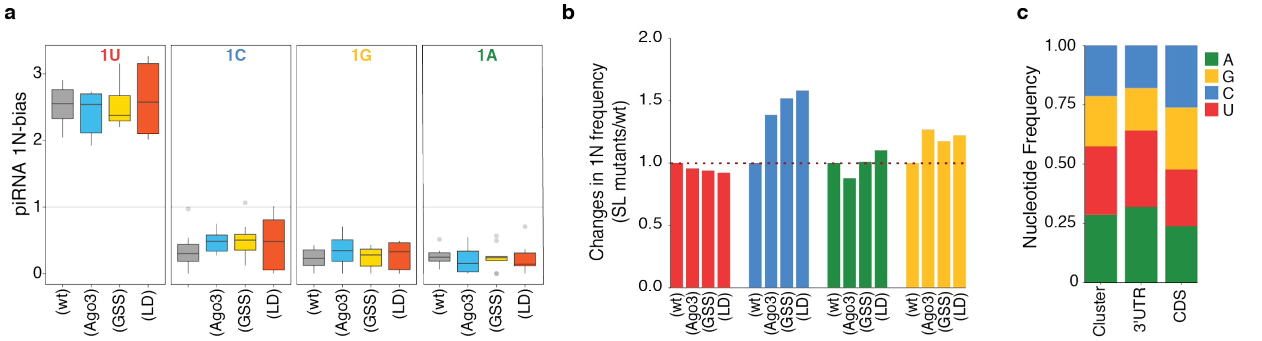

Supplementary Fig. 4. First nucleotide bias for piRNAs from piRNA clusters and structural models of Piwi's SL in wt and SL mutants. a The nucleotide bias at the first position (piRNA 1N-bias) of piRNAs is calculated as observed piRNA 5' nucleotide frequency per expected nucleotide frequency for well-represented piRNA clusters (75th percentile by total piRNA abundance). PiRNAs that are associated with FH-Piwi(wt) and two SL-mutants, -SL(Ago3), -SL(GSS) and -SL(LD) in fly ovaries. **b** Change in 1N frequency for all cluster piRNAs in mutants compared to wild type in fly ovaries. **c** Comparison of mean nucleotide composition of piRNA clusters, 3´ UTRs and protein coding regions.

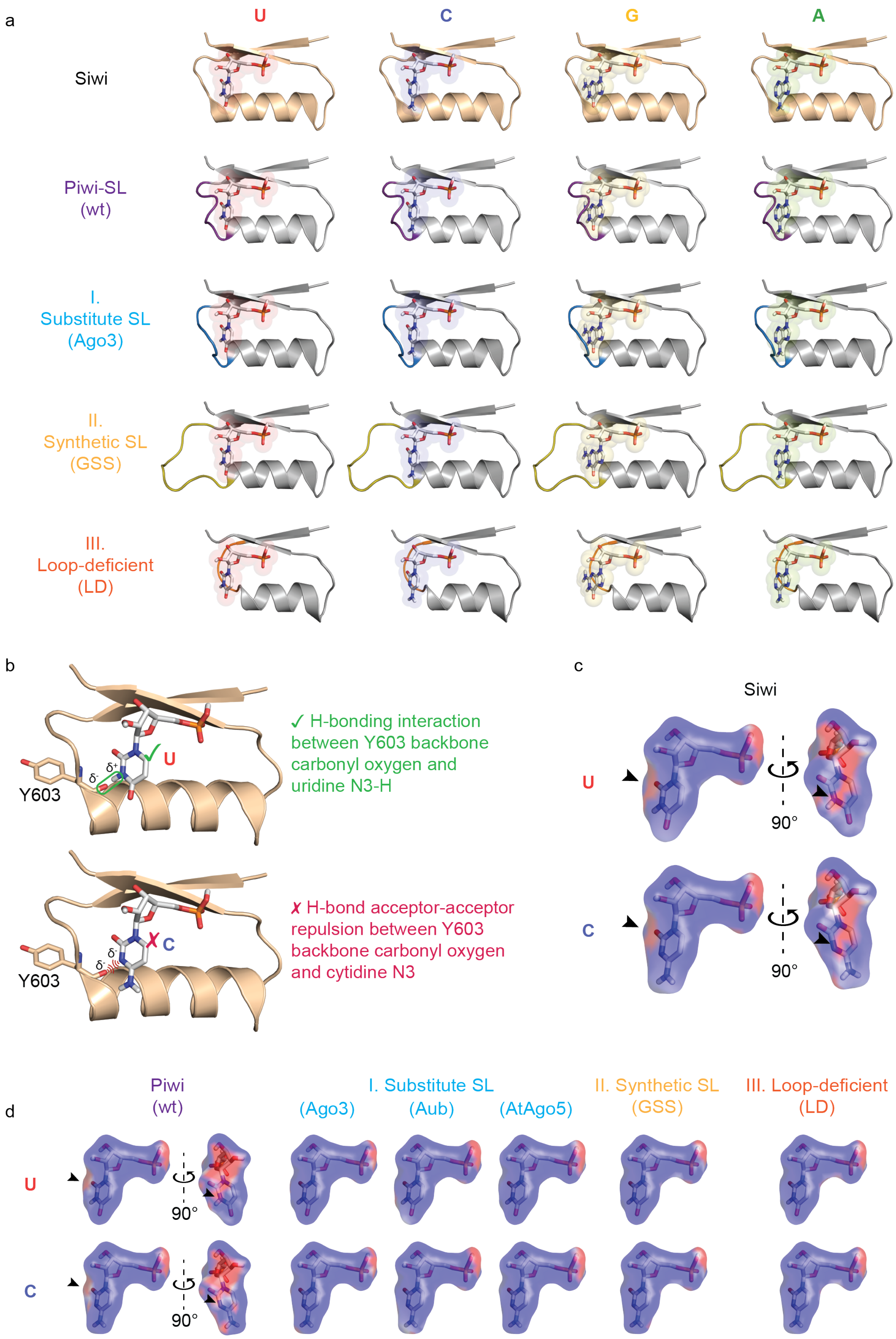

a
U
C
G
A
Siwi
Piwi-SL (wt)
I. Substitute SL (Ago3)
II. Synthetic SL (GSS)
III. Loop-deficient (LD)
b
Y603
U
δ+
δ-
✓ H-bonding interaction between Y603 backbone carbonyl oxygen and uridine N3-H
Y603
C
δ-
δ-
✗ H-bond acceptor-acceptor repulsion between Y603 backbone carbonyl oxygen and cytidine N3
c
Siwi
U
90°
C
90°
d
Piwi (wt)
I. Substitute SL
(Ago3)
(Aub)
(AtAgo5)
II. Synthetic SL (GSS)
III. Loop-deficient (LD)
U
90°
C
90°

42

Supplementary Fig. 5. Structural models and predicted interactions of the Piwi specificity loop. a Structural models were generated for each Piwi protein sequence. The overall structure of each protein construct was first estimated by homology modeling, which was then followed by refinement of the specificity loop (see Molecular Modeling section of Methods). For each model (indicated left), the protein region surrounding the 5' nucleotide (indicated top) is shown. The corresponding region from the Siwi crystal structure is presented for reference (top row). Specificity loops are colored as notated in Figure 1 (wildtype: purple, Ago3: light blue, GSS: dark yellow, LD: orange). For the Piwi models, as predicted, the wildtype and Ago3 loops most closely resemble that in the Siwi structure whereas the GSS and loop deletion mutants dramatically alter the SL conformation. **b** In the structure of Siwi bound to RNA (top), the authors noted an H-bonding interaction between Tyr603 and the uridine base (green, upper panel). If in the same location, cytidine is predicted to be repelled due to adjacent, partially negatively-charged, H-bond acceptors (red, lower panel). **c** Mapping of the electrostatic potential calculated from the Siwi structure onto the surface of uridine or cytidine predicts that the repulsive effect between partially negatively-charged atoms indicated in **b** would be exacerbated by the local electrostatic environment (arrowheads). **d** Mapping of the electrostatic potentials calculated for the Piwi protein models (labeled top) predicts a similar charge distribution to **c** for the wildtype specificity loop, but not for the specificity loop mutants. [The electrostatic surfaces displayed in **c** and **d** are generated *from* the protein models and applied *to* the nucleotide surface. The surfaces are not the electrostatic maps of the nucleotides themselves.]

Supplementary Data:

Supplementary Data 1: Plasmid and oligonucleotides

Supplementary Data 2: Mapping statistics for Illumina libraries : OSC sample (Sheet 1); Fly ovary samples (Sheet 2). Information on Unique mappers (M=1) and Multi mappers (M<=100). Is provided for each sequencing sample.

Supplementary Data 3: Count tables used for analyses in Figure 1. (Supplementary Data 3 contains count information used to generate all the plots in Figure 1 from OSC samples.) Size Distribution (Sheet 1): read counts of all the mapped reads for a single replicate of 10 FH-Piwi_SL samples. TE targeting (Sheet 2): read counts of all 24-29-nt long reads mapped to antisense of transposon families. Genomic annotation (Sheet3): read counts of all 24-29-nt long reads mapped to sense and antisense of all transposons, exons and introns. 1st Nucleotide frequencies (Sheet 4): 1st nucleotide counts for all 24-29-nt reads.

Supplementary Data 4: Count tables used for analyses in Figure 2**.** (Supplementary Data 4 contains count information used to generate all the plots in Figure 2 from Fly ovary samples.) Size Distribution (Sheet 1): read counts of all the mapped reads for a single replicate of 4 FH-Piwi_SL samples. TE targeting (Sheet 2): read counts of all 24-29-nt long reads mapped to antisense of transposon families. Genomic annotation (Sheet3): read counts of all 24-29-nt long reads mapped to sense and antisense of all transposons, exons and introns. 1st Nucleotide frequencies (Sheet 4): 1st nucleotide counts for all 24-29-nt reads.

Supplementary Data 5: Count tables used for analyses in Figure 3 and Supplementary Figure 3. (Supplementary Data 5 contains count information used to generate all the metagene plots in Figure 3 and Supplementary Figure 3 for OSC, Fly ovary and Mouse testis samples.) OSC metagene (Sheet 1): nucleotide counts for every position in 101-nt window generated using unique

24-29-nt long reads for OSC samples. Data for individual replicate. Fly ovary metagene (Sheet 2): nucleotide counts for every position in 101-nt window generated using unique 24-29-nt long reads for Fly ovary samples. Mouse metagene (Sheet 3): nucleotide counts for every position in 101-nt window generated using all unique reads for mouse Miwi sample (SRR5304346).

Supplementary Data 6: Count tables used for analyses in Figure 4 and Supplementary Figure 4 (Supplementary Data 6 contains count information for the OCS and Fly ovary clusters used for plots in Figure 4 and Supplementary Figure 4.) OSC (Sheet 1): cluster information and 1st nucleotide counts using unique 24-29-nt long reads for OSC samples. Data for individual replicate. Fly ovary (Sheet 2): cluster information and 1st nucleotide counts using unique 24-29-nt long reads for Fly ovary samples
