# Supplementary material for: Decoding the 5′ nucleotide bias of PIWI-interacting RNAs
Source: Nat Commun. 2019 Feb 19;10:828. doi: 10.1038/s41467-019-08803-z (PMC6381166; doi:10.1038/s41467-019-08803-z)
Supplement: Supplementary file 2 — Description of Additional Supplementary Files [file 41467_2019_8803_MOESM2_ESM.docx]

**Description of Additional Supplementary Files**

File Name: Supplementary Data 1

Description: Plasmid and oligonucleotides.

File Name: Supplementary Data 2

Description: Mapping statistics for Illumina libraries: OSC sample (Sheet 1); Fly ovary samples (Sheet 2). Information on Unique mappers (M=1) and Multi mappers (M<=100). Is provided for each sequencing sample.

File Name: Supplementary Data 3

Description: Count tables used for analyses in Figure 1. (Supplementary Data 3 contains count information used to generate all the plots in Figure 1 from OSC samples.) Size Distribution (Sheet 1): read counts of all the mapped reads for a single replicate of 10 FH-Piwi_SL samples. TE targeting (Sheet 2): read counts of all 24-29-nt long reads mapped to antisense of transposon families. Genomic annotation (Sheet3): read counts of all 24-29-nt long reads mapped to sense and antisense of all transposons, exons and introns. 1st Nucleotide frequencies (Sheet 4): 1st nucleotide counts for all 24-29-nt reads.

File Name: Supplementary Data 4

Description: Count tables used for analyses in Figure 2. (Supplementary Data 4 contains count information used to generate all the plots in Figure 2 from Fly ovary samples.) Size Distribution (Sheet 1): read counts of all the mapped reads for a single replicate of 4 FH-Piwi_SL samples. TE targeting (Sheet 2): read counts of all 24-29-nt long reads mapped to antisense of transposon families. Genomic annotation (Sheet3): read counts of all 24-29-nt long reads mapped to sense and antisense of all transposons, exons and introns. 1st Nucleotide frequencies (Sheet 4): 1st nucleotide counts for all 24-29-nt reads.

File Name: Supplementary Data 5

Description: Count tables used for analyses in Figure 3 and Supplementary Figure 3. (Supplementary Data 5 contains count information used to generate all the metagene plots in Figure 3 and Supplementary Figure 3 for OSC, Fly ovary and Mouse testis samples.) OSC metagene (Sheet 1): nucleotide counts for every position in 101-nt window generated using unique 24-29-nt long reads for OSC samples. Data for individual replicate. Fly ovary metagene (Sheet 2): nucleotide counts for every position in 101-nt window generated using unique 24-29-nt long reads for Fly ovary samples. Mouse metagene (Sheet 3): nucleotide counts for every position in 101-nt window generated using all unique reads for mouse Miwi sample (SRR5304346)

File Name: Supplementary Data 6

Description: Count tables used for analyses in Figure 4 and Supplementary Figure 4 (Supplementary Data 6 contains count information for the OCS and Fly ovary clusters used for plots in Figure 4 and Supplementary Figure 4.) OSC (Sheet 1): cluster information and 1st nucleotide counts using unique 24-29-nt long reads for OSC samples. Data for individual replicate. Fly ovary (Sheet 2): cluster information and 1st nucleotide counts using unique 24-29-nt long reads for Fly ovary samples
